# Access to rehabilitation after stroke in Brazil (AReA study): multicenter study protocol

**DOI:** 10.1055/s-0042-1758558

**Published:** 2022-12-19

**Authors:** Roberta de Oliveira Cacho, Carla Heloisa Cabral Moro, Rodrigo Bazan, Suzete Nascimento Farias da Guarda, Elen Beatriz Pinto, Suellen Mary Marinho dos Santos Andrade, Lenise Valler, Kelson James Almeida, Tatiana Souza Ribeiro, Renata Viana Brígido de Moura Jucá, Cesar Minelli, Maria Elisa Pimentel Piemonte, Eric Homero Albuquerque Paschoal, Marco Túlio Araújo Pedatella, Octávio Marques Pontes-Neto, Ana Paula Fontana, Aline de Souza Pagnussat, Adriana Bastos Conforto, AReA Study Group

**Affiliations:** 1 Universidade Federal do Rio Grande do Norte, Faculdade de Ciências da Saúde do Trairi, Santa Cruz RN, Brazil.; 2 Hospital Municipal de São José, Joinville SC, Brazil.; 3 Universidade Estadual Paulista, Faculdade de Medicina de Botucatu, São Paulo SP, Brazil.; 4 Universidade Federal da Bahia, Departamento de Neurociências e Saúde Mental, Faculdade de Medicina da Bahia, Salvador BA, Brazil.; 5 Escola Bahiana de Medicina e Saúde Pública, Fundação para o Desenvolvimento das Ciências, Salvador BA, Brazil.; 6 Universidade Federal da Paraíba, Departamento de Fisioterapia, João Pessoa PB, Brazil.; 7 Universidade Estadual de Campinas, Faculdade de Ciências Médicas, Campinas SP, Brazil.; 8 Universidade Federal do Piauí, Centro Universitário UniFacid, Departamento de Medicina Especializada em Neurologia, Teresina PI, Brazil.; 9 Universidade Federal do Rio Grande do Norte, Departamento de Fisioterapia, Natal RN, Brazil.; 10 Universidade Federal do Ceará, Hospital Universitário Walter Cantídio, Fortaleza CE, Brazil.; 11 Hospital Carlos Fernando Malzoni, Instituto “Você sem AVC”, Matão SP, Brazil.; 12 Universidade de São Paulo, Departamento de Neurociências e Ciências Comportamentais, Faculdade de Medicina de Ribeirão Preto, Ribeirão Preto SP, Brazil.; 13 Universidade de São Paulo, Faculdade de Medicina, Departamento de Fisioterapia, Fonoaudiologia e Terapia Ocupacional, São Paulo SP, Brazil.; 14 Hospital Ophir Loyola, Belém PA, Brazil.; 15 Hospital Estadual de Urgência de Goiânia Dr Valdemiro Cruz, Goiânia GO, Brazil.; 16 Universidade de São Paulo, Faculdade de Medicina de Ribeirão Preto, Ribeirão Preto SP, Brazil.; 17 Universidade Federal do Rio de Janeiro, Faculdade de Fisioterapia, Laboratório Pesquisa em Recuperação Funcional Após AVC, Rio de Janeiro RJ, Brazil.; 18 Universidade Federal de Ciências da Saúde de Porto Alegre, Departamento de Fisioterapia, Porto Alegre RS, Brazil.; 19 Universidade de São Paulo, Hospital de Clínicas, Divisão de Neurologia Clínica, São Paulo SP, Brazil.; 20 Hospital Israelita Albert Einstein, São Paulo SP, Brazil.

**Keywords:** Stroke, Patient Discharge, Referral and Consultation, Rehabilitation, Acidente Vascular Cerebral, Alta do Paciente, Encaminhamento e Consulta, Reabilitação

## Abstract

**Background** Most of the Brazilian population relies on public healthcare and stroke is a major cause of disability in this country of continental dimensions. There is limited information about access to rehabilitation after stroke in Brazil.

**Objective** To provide comprehensive information about Access to Rehabilitation After discharge from public hospitals in Brazil (AReA study), up to 6 months after stroke.

**Methods** The present study intends to collect information from 17 public health centers in 16 Brazilian cities in the 5 macroregions of the country. Each center will include 36 participants ( *n* = 612). The inclusion criteria are: age ≥ 18 years old; ischemic or hemorrhagic stroke, from 6 months to 1 year prior to the interview; admission to a public hospital in the acute phase after stroke; any neurological impairment poststroke; patient or caregiver able to provide informed consent and answer the survey. Patients can only be recruited in public neurology or internal medicine outpatient clinics. Outcomes will be assessed by a standard questionnaire about rehabilitation referrals, the rehabilitation program (current status, duration in months, number of sessions per week) and instructions received. In addition, patients will be asked about preferences for locations of rehabilitation (hospitals, clinics, or at home).

**Trial Status** The study is ongoing. Recruitment started on January 31 ^st^ , 2020 and is planned to continue until June 2022.

**Conclusion** The AReA study will fill a gap in knowledge about access to stroke rehabilitation in the public health system in different Brazilian regions.

## Resumo

**Antecedentes** Grande parte da população brasileira depende de saúde pública e o acidente vascular cerebral (AVC) é uma das principais causas de incapacidade neste país de dimensões continentais. As informações sobre o acesso à reabilitação após AVC em instalações públicas no Brasil são limitadas.

**Objetivo** Fornecer informações abrangentes sobre o Acesso à Reabilitação pós-AVC (estudo AReA) nos primeiros 6 meses após a alta hospitalar da rede pública.

**Métodos** Serão coletadas informações de 17 centros de saúde públicos em 16 cidades brasileiras das cinco macrorregiões do país. Cada centro incluirá 36 participantes ( *n* = 612). Os critérios de inclusão são: idade ≥ 18 anos; AVC isquêmico ou hemorrágico, com tempo de lesão entre 6 meses e 1 ano; admissão em hospital público na fase aguda; qualquer comprometimento neurológico pós-AVC; paciente ou cuidador capaz de fornecer consentimento informado e responder à pesquisa. Os pacientes só podem ser recrutados em ambulatórios públicos de neurologia ou medicina interna. Os resultados serão avaliados por um questionário padrão sobre encaminhamentos de reabilitação, o programa de reabilitação (estado atual, duração em meses, número de sessões por semana) e instruções recebidas. Além disso, os pacientes serão questionados sobre as preferências de locais de reabilitação (hospitais, clínicas ou casa).

**Status do estudo** O estudo está em andamento. O recrutamento começou em 31 de janeiro de 2020 e está previsto para continuar até junho de 2022.

**Conclusão** O estudo AReA preencherá uma lacuna no conhecimento sobre o acesso à reabilitação para AVC no sistema público de saúde em diferentes regiões brasileiras.

## INTRODUCTION

According to the World Health Organization (WHO), rehabilitation is a set of interventions designed to optimize functioning and reduce disability in individuals with health conditions in interaction with their environment. It is a core health service for individuals throughout their life course, across the continuum of care for a range of acute and chronic health conditions, among them, poststroke patients. 1

Current guidelines recommended that subjects with stroke should be assessed by a multidisciplinary team within 24 to 48 hours after admission and referred to a rehabilitation program immediately after discharge. 2 According to the INTERSTROKE study, 3 which compared the standard of care available for 13,447 poststroke patients in 32 countries between 2007 and 2015, access to rehabilitation after hospital discharge was associated with less severe dependence and greater probability of survival at 1 month post-stroke. The study identified a great need for research focusing on inclusion and early transition to rehabilitation, especially in low- and middle-income countries.

Despite many gaps in knowledge, there is some evidence that rehabilitation is insufficient for patients with stroke in different parts of the world. 4 5 6 7 8 Greater availability of rehabilitation and functional improvements are found in high-income countries, while low- and middle-income countries continue to face several barriers 9 such as restrictions on transportation, long waiting times, limited information, lack of referral at/after discharge, lack of community or social support, ineffective communication with health professionals, inadequate discharge planning, and lack of knowledge and awareness of the benefits of outpatient rehabilitation. 5 9 10

For instance, while a Canadian study 10 showed that 59.5% of the patients with stroke were referred to at least 1 rehabilitation service after discharge and 81% received rehabilitation, only 14% of South African subjects were found to have access to outpatient treatment. 11 A systematic review about stroke care in Africa identified limited access to medical and physical therapy services in the continent, likely due to costs and geographic inaccessibility. 6 Likewise, a study in India revealed the availability of only 2 rehabilitation centers in a state with more than 70 million inhabitants. 12

Even in developed countries, the rehabilitation process often does not follow evidence-based practices and is not conducted by a multidisciplinary team. 13 There are some reasons for this, such as the lack of training in multidisciplinary team work as well as perceptions that no rehabilitation is required for patients with minor deficits. 14 Support for self-management postdischarge and information about stroke care were the main challenges identified in a study about perceived quality of transition care conducted in Sweden. 15

Some barriers to rehabilitation are shared by low- or middle-income and high-income countries. Referral delays (50%) and difficulties in transportation/distance to healthcare facilities (39%) were reported by 239 responders to surveys in Peru and in the United States of America as the main obstacles for rehabilitation. 16 Geographic access is a major challenge to adherence to treatment. Being in rural areas at large distances from health services negatively impacts the rehabilitation process. 17

In addition, patients with mild or severe impairments tend to be less frequently referred to rehabilitation services. 10 18 Being independent within 72 hours of admission or having dementia can be negatively associated with assessment of the presence of rehabilitation needs. 4 On the other hand, a higher level of education is related to greater participation in outpatient rehabilitation in the United States. 5

In Brazil, stroke is a leading cause of death and disability. 19 According to the National Health Survey (PNS) in 2013, the point prevalence of stroke were 1.6 and 1.4% in men and women, respectively, while the prevalence of poststroke disabilities was 29.5% for men and 21.5% for women. 20 According to the National Health Survey (PNS) in 2013, stroke prevalence was 2.9% in the age group between 60 and 64 years old and 7.3% in subjects > 75 years old. 20 Limited information is available about access to rehabilitation in the Brazilian Unified Health System (SUS, in the Portuguese acronym), even though three-quarters of the Brazilian population relies exclusively on this system and one-quarter uses both public and private care. 21 22 Rehabilitation needs after stroke are likely to become a bigger problem in the future considering that, over the next years, the number of subjects with stroke is expected to grow due to the rapid ageing of the Brazilian population.

For instance, in a university hospital in São Paulo, the largest Brazilian city, 70% of 665 patients followed-up for 4 years after a first-ever stroke did not receive any type of rehabilitation after discharge. These patients had low socioeconomic and educational levels, in addition to high severity of neurological impairments. 22 In 2016, the Scientific Department of Neurorehabilitation from the Brazilian Academy of Neurology (SCD-BAN) conducted a national survey about access to post-stroke rehabilitation. A total of 370 neurologists answered the survey and 80.2% considered that access was inadequate. 24

Identifying barriers to access the rehabilitation interventions is crucial to improve the health system integration and the resources required to deliver them safely and effectively. Such information would contribute to strengthening health systems in rehabilitation, guiding health policies, planning, and budgeting.

Considering the limited available evidence about access to rehabilitation after stroke in Brazil, SCD-BAN invited neurologists and rehabilitation professionals from five different Brazilian regions to participate in a comprehensive observational study – AReA (Access to post-stroke REhabilitation After stroke in Brazil). The main objective of the present study is to provide quantitative information about access to rehabilitation after discharge from public hospitals, up to 6 months after stroke. Secondary objectives are to assess predictors of access to rehabilitation, and the association between rehabilitation and functional outcomes.

## METHODS

### Study design

In the present multicenter study, we aim to include 612 patients between 6 months and 1 year after ischemic or hemorrhagic stroke.

### Ethics approval and consent to participate

The final study protocol and written informed consent were approved by the Albert Einstein Hospital Ethics Committee (Protocol 4.080.346/2020; CAAE: 19110619.7.1001.0071) and by Ethics Committees of each participating center. Written informed consent must be obtained either from the patient or his/her family member through the SurveyMonkey platform. Progress reports are submitted to the coordinating center Ethics Committee, every 6 months.

### Study population

#### *Inclusion and exclusion criteria*

The inclusion criteria are: age ≥ 18 years old; ischemic or hemorrhagic stroke, from 6 months to 1 year prior to the interview; admission to a public hospital in the acute phase after stroke; any neurological impairment poststroke; patient or caregiver able to provide informed consent and answer the survey. Patients with other neurological conditions such as Parkinson or Alzheimer disease are excluded. Patients can only be recruited in public neurology or internal medicine outpatient clinics, not in rehabilitation facilities or clinics, in order to avoid bias.

#### *Recruitment strategy*

**Figure 1:**
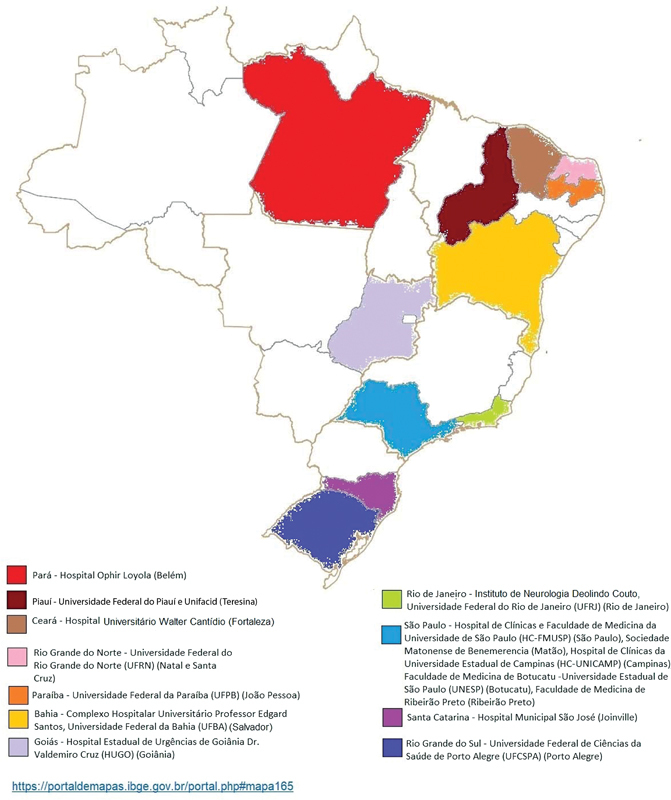
Participating centers in Brazil.

The participants are invited to participate after regular outpatient visits in 17 centers distributed in 11 states across Brazil ( Figure 1 ). Recruitment started on January 31 ^st^ , 2020 and is planned to continue until June 2022. The first case of COVID-19 was diagnosed in Brazil in February 2020 25 and, until that date, only 2 participants had been included in the present study. Therefore, most participants are expected to be included during the pandemic.

#### *Characteristics of the patients*

The following data are collected: gender, age, educational level, time from stroke, type (ischemic or hemorrhagic), lesion location, number of strokes, performance of computed tomography (CT) or magnetic resonance imaging (MRI) for diagnosis, National Institutes of Health Stroke Scale (NIHSS) 26 and modified Rankin Scale (mRS) scores. 27

### Outcomes

The primary outcome is the percentage of patients who had access to any physical therapy session after hospital discharge within the first 6 months after stroke onset.

The secondary outcomes are: referral to physical therapy after hospital discharge, regardless of actual access; the percentages of patients who had access to each of the following professionals after discharge: occupational therapist, speech therapist, neurologist, psychologist, physiatrist and nutritionist; the percentage of patients who received health instructions until 6 months after stroke; preferences of patients and families regarding the rehabilitation process (home-based, outpatient, inpatient services).

Outcomes will be assessed by a standard questionnaire ( Table 1 ).

**Table 1:** Access to rehabilitation questionnaire

**Questions related to referrals to rehabilitation**		
1	In the first 6 months after hospital discharge due to stroke, were you treated by a **physiotherapist** *?	(__) Yes (__) No (__) Unknown
2	Has a treatment program been established by a **physiotherapist** *?	(__) Yes (__) No
3	What is the current situation of the physical therapy* program?	(__) Finished (__) In progress (__) I don't know
4	How was the physical therapy* program distributed?	(__) Duration in months (__) Number of sessions per week
5	What is the establishment/institute where the physical therapy* program was carried out?	(_____________) free answer
*Questions 1 to 5 are then repeated. Words with asterisks are replaced by: physiatrist, speech therapist, neurologist, psychologist, occupational therapist, and nutritionist.		
**Questions related to access to rehabilitation, treatments, and orientations**		
6	On discharge, have you been referred to any rehabilitation service?	(__) No (stroke with neurological impairments but no referral was done) (__) No (stroke without neurological impairments) (__) Yes. What kind?_____________
7	If you were referred to rehabilitation: did you have access to the rehabilitation professional or service to which you were referred?	(__) No (__) Yes. For how long?______________
8	Did you have to search for a rehabilitation service on your own because you were not referred by a health professional?	(__) No (__) Yes. What kind?_____________
9	If you had to search for a rehabilitation service on your own, were you able to receive rehabilitation?	(__) No (__) Yes. For how long?______________
10	Did you receive any instructions about upper limb positioning after stroke?	(__) No, I did not receive any guidance despite my upper limb paresis (__) No, I did not receive any guidance because my upper limb is not affected after stroke (__) Yes, during hospitalization (__) Yes, in (__) weeks after discharge
11	Did you receive any instructions about your upper limb movements after stroke?	(__) No, I did not receive any guidance despite my upper limb paresis (__) No, but my upper limb was not affected after stroke (__) Yes, during hospitalization (__) Yes, in (__) weeks after discharge
12	Did you receive instructions about the possibility to eat any kind of food after stroke?	(__) No, I didn't receive any guidance (__) Yes, during hospitalization (__) Yes, in (__) weeks after discharge
13	Did you receive any instructions about physical activity after stroke?	(__) No, I didn't receive any guidance (__) Yes, during hospitalization (__) Yes, in (__) weeks after discharge
14	If you need rehabilitation, where would you like it to be carried out?	(__) Inpatient hospital (__) Rehabilitation centers, even though far from my home (__) In a health center, nearby my home (__) Home-based rehabilitation, carried by a professional (__) Home-based rehabilitation, carried by a family member
15	Would you like to receive instructions or supervision about exercises to do at home (to improve your arm or leg, for example), in a smartphone?	(__) Yes (__) No

The first part is related to rehabilitation referrals that the participant received within the first 6 months after hospital discharge and the rehabilitation program (current status, duration in months, number of sessions per week). The second part is related to instructions received (such as upper limb positioning and movements, feeding, physical activity) and the preference of the participant (treatment in hospitals, rehabilitation centers, or at home).

### Study procedures

Prior to recruitment, all researchers involved in data collection are required to receive online certification in assessment of the NIH stroke scale (NIHSS - http://nihss-portuguese.trainingcampus.net ) and the modified Rankin Scale (mRS - https://forms.gle/KqChiJvRNsfGe5oU6 ) scores.

Training in ethical principles and clinical research is also required. Some courses in English or Portuguese are suggested ( https://edx.hospitalmoinhos.org.br/course/etica-em-pesquisa-clinica ; https://gcp.nidatraining.org/ ; - http://www.aagapesantamarcelina.com.br/ead/login/index.php ).

Once certifications are completed, the Principal Investigator (PI) from each center receives a training link to the questionnaire registered on the SurveyMonkey platform. He or she practices the interviews with the researchers involved in data collection. Then, the PI receives the link for the questionnaire and a Microsoft Excel, version 2210, 64 bits, (Microsoft Corporation, Redmond, WA, USA) template to register the data.

The questionnaire can be answered by the patient/participant or, if impossible due to language barriers, by a proxy. The interview lasts ∼ 20 minutes.

### Data monitoring

The local PI is responsible for supervising data collection, storage, and transfer to the coordinating center. Conforto A. B. and ROC oversee the study. Every month, local PIs receive a newsletter with information about the progress of the project, deadlines, and instructions. Data are anonymously stored on the SurveyMonkey platform.

### Ethics

The present study was approved by the Human Research Ethics Committee of Albert Einstein Hospital (CAAE: 19110619.7.1001.0071), the coordinating center, and by local ethics committees from participating centers. All participants or proxies must provide informed consent through the SurveyMonkey platform. Progress reports are submitted to the coordinating center Ethics Committee every 6 months.

### Sample size

Sample size was not formally calculated in the present observational, exploratory study. We plan to include 612 participants representative of patients from the five main Brazilian regions.

### Statistical analyses

Descriptive statistical analysis will be performed for all outcomes in each center and in the total sample.

Chi-squared tests will be performed to compare the following variables according to access or lack of access to each professional involved in rehabilitation care: age, gender, area (North/Northeastern/Western states of Brazil versus South/Southeastern states), NIHSS and timing of data collection (in months, after January 2020).

Three multivariate logistic regression models will be performed to identify independent predictors of access to rehabilitation in the overall sample. The dependent variables will include access to any type of professional involved in rehabilitation; access to physical, occupational, and speech therapy; access to all professionals (occupational therapist, speech therapist, neurologist, psychologist, physiatrist, and nutritionist). The independent variables will include region, age, gender, and type of stroke.

Percentages of access to physical therapists and access to other professionals across each region will be compared with chi-squared tests.

In addition, an exploratory logistic regression analysis of the association between the amount of time spent in all rehabilitation sessions (physical, occupational or speech therapy) and mRS scores (≥ 2 or < 2) will be performed. Age, gender, and NIHSS score will be included as covariates.

P-values ≤ 0.05 will be considered statistically significant. The program that will be performed the statistical test will be JASP statistics, version 0.14.3 Eric-Jan Wagenmakers (room G 0.29) Department of Psychological Methods University of Amsterdam Nieuwe Achtergracht 129B Amsterdam, The Netherlands.

## DISCUSSION

One of the national priorities in the fight against stroke in Brazil, a Latin-American country of continental dimensions is to structure rehabilitation services and home care. 28 The WHO Rehabilitation 2030 initiative draws attention to the profound unmet need for rehabilitation worldwide and highlights the importance of strengthening health systems to provide rehabilitation, determining that rehabilitation should be available for all the population and through all stages of the life course. 1 The Gramado declaration aimed to provide strategies to reduce the burden of stroke in Latin America and recommended increased access to rehabilitation. However, data about poststroke care are still scarce in the region. 8 The AReA study will fill a major gap in knowledge about access to rehabilitation in the public health system in different Brazilian regions.

Physical rehabilitation is a key intervention after stroke in low- and lower-middle income countries, improving outcomes such as functional ability, walking capacity, and balance. 29 A session of 30 to 60 minutes per day of physical rehabilitation delivered by a physiotherapist, physical therapist, or rehabilitation therapist during 5 to 7 days per week is considered to be effective. 30 Outpatient and/or in-home rehabilitation services should last at least 8 weeks (Evidence level C). 31 However, in clinical practice, the duration and intensity of rehabilitation are often insufficient. In Brazil, physical therapy is often delivered in two sessions per week, but protocols for discharge from the rehabilitation process are scarce.

In the present study, access to physical therapy was chosen as the main outcome because of a perception that, in clinical practice, this is the most widely available rehabilitation intervention in Brazil. We hypothesize that occupational therapy, speech therapy, physiatry consults or psychological support are less often offered by the SUS. This study will address this hypothesis and allow comparisons between different Brazilian regions.

The questionnaire in the AreA study addresses some particular issues: upper limb rehabilitation, because more than half of stroke survivors continue to have upper limb disability for months to years 32 ; speech therapy, since dysphagia due to stroke can occur in up to 67% of cases and influences prognosis 13 ; instructions about physical activity because it is recommended that rehabilitation programs promote a reduction in sedentary time and enhance level of physical activity to decrease the risk of recurrent stroke and other cardiovascular diseases. 33

The first case of SARS-CoV2 infection was identified in Brazil on February 26 ^th^ , 2020 25 and, until then, only 2 patients had been included in AReA. The COVID-19 pandemic imposed dramatic burdens on health systems in general, and neurorehabilitation in particular, worldwide. 34 In the south of Brazil, stroke admissions in a stroke center decreased by 36.4% in 2020, when compared with the same period in 2019. It is possible that patients feared contamination by SARS-CoV2 in hospitals. 35 Delays in stroke diagnosis or treatment may lead to increased risk of recurrence and disability. 35 In addition, many outpatient clinics were shut down or operated with limited personnel to treat a small fraction of patients, compared with the prepandemic era. Group therapy was interrupted to avoid cross-contamination of patients and therapists. Overall, these changes are expected to deepen a preexistent abyss of barriers to rehabilitation and strongly impact the results of the present study.

Until now, telerehabilitation is not widely available in Brazil. No evidence-based systems are available in the SUS. Internet use in the country is one of the greatest in the world but access is limited in remote areas. 36 Furthermore, family members in home office may not be available to assist patients so that they can virtually connect with therapists. The data collected in the AReA study are expected to contribute to understand if alternatives to face-to-face rehabilitation were or not available in Brazil during the pandemic, and to increase knowledge about preferences of the patients regarding rehabilitation delivered at hospitals, rehabilitation centers, or at home, in the novel scenario of a global pandemic. Studies in lower-resourced areas are required, especially for investigations about specific needs, existing infrastructure, and potential barriers for telerehabilitation, self-rehabilitation, and community-based rehabilitation. 29 In addition, future studies about access to rehabilitation in particular subgroups of patients with stroke (for instance, with other diseases that can lead to heart disease, such as Chagas disease and coronary heart disease) are needed. 37 38

In summary, AReA will provide a snapshot of rehabilitation services in Brazil and most of the data will be collected during the COVID-19 pandemic. The results of the present study will be key to develop public health strategies and to guide efforts to provide novel rehabilitation options in a challenging context, to change the game against disability from stroke.
